# Experimental Approaches to Study Genome Packaging of Influenza A Viruses

**DOI:** 10.3390/v8080218

**Published:** 2016-08-09

**Authors:** Catherine Isel, Sandie Munier, Nadia Naffakh

**Affiliations:** 1 Architecture et Réactivité de l’ARN, Université de Strasbourg, Centre National de la Recherche Scientifique (CNRS), Institut de Biologie Moléculaire et Cellulaire (IBMC), 15 rue René Descartes, 67084 Strasbourg, France; 2 Département de Virologie, Unité de Génétique Moléculaire des Virus à ARN, Institut Pasteur, 75015 Paris, France; sandie.munier@pasteur.fr; 3 Centre National de la Recherche Scientifique (CNRS), Unité Mixte de Recherche 3569, 75016 Paris, France; 4 Unité de Génétique Moléculaire des Virus à ARN, Sorbonne Paris Cité, Université Paris Diderot, 75013 Paris, France

**Keywords:** influenza virus, packaging signal, packaging assay, single-molecule FISH, RNA-RNA interaction, competitive reverse genetics

## Abstract

The genome of influenza A viruses (IAV) consists of eight single-stranded negative sense viral RNAs (vRNAs) encapsidated into viral ribonucleoproteins (vRNPs). It is now well established that genome packaging (i.e., the incorporation of a set of eight distinct vRNPs into budding viral particles), follows a specific pathway guided by segment-specific *cis*-acting packaging signals on each vRNA. However, the precise nature and function of the packaging signals, and the mechanisms underlying the assembly of vRNPs into sub-bundles in the cytoplasm and their selective packaging at the viral budding site, remain largely unknown. Here, we review the diverse and complementary methods currently being used to elucidate these aspects of the viral cycle. They range from conventional and competitive reverse genetics, single molecule imaging of vRNPs by fluorescence in situ hybridization (FISH) and high-resolution electron microscopy and tomography of budding viral particles, to solely in vitro approaches to investigate vRNA-vRNA interactions at the molecular level.

## 1. Introduction

**Figure 1:**
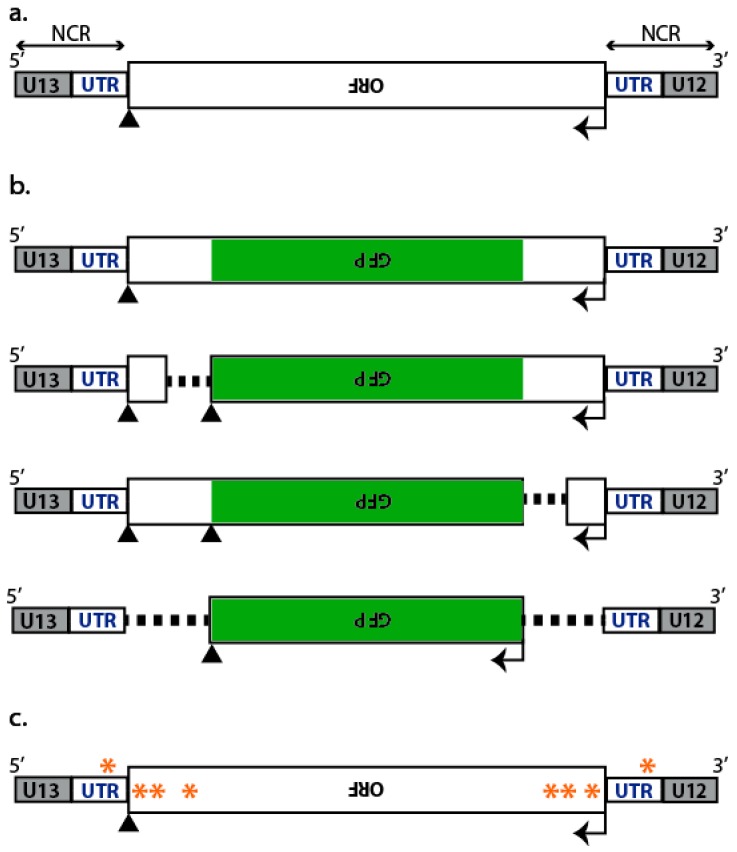
Schematic diagram of influenza A viral RNA segments (vRNAs). (**a**) Genetic organization of the influenza A virus (IAV) genomic segments. Each segment contains at least one open reading frame (ORF) in antisense orientation, flanked by segment specific non-coding regions (NCRs) encompassing untranslated regions (UTR) and conserved unique (U) promoter regions of 12 (U12) and 13 (U13)-nucleotides (nt) long; (**b**) Schematic diagram of green fluorescent protein (GFP)-reporter vRNAs. The center portion of the coding region is replaced with the GFP coding region (green), flanked by portions of various lengths of the corresponding termini of the coding region of interest (white); (**c**) Schematic representation of vRNAs bearing synonymous mutations (orange asterisks) introduced into the NCRs and termini of the coding regions. Initiation and stop codons are indicated by arrows and triangles, respectively.

Influenza A viruses (IAVs) are responsible for yearly flu epidemics that cause three to five million cases of severe illness, claim 250,000 to 500,000 lives annually and greatly impact the global economy. Increased morbidity and mortality can also result from occasional pandemics, the latest one being in 2009. IAVs are members of the *Orthomyxoviridae* family. Their genome consists of eight single-stranded negative sense viral RNA segments (vRNAs), varying in length from 2341 to 890 nucleotides (nt), numbered from 1 to 8 or named after the main protein they encode. Despite their difference in length, all vRNAs share the same genetic organization: the functional open reading frame (ORF), in antisense orientation, is flanked by two non-coding regions (NCRs) that differ in length and in sequence between vRNAs, except for the 12- and 13-nt long sequences at the 3′ and 5′ end, respectively, that are highly conserved between vRNAs and between species (Figure 1a). These sequences are partially complementary and anneal to form a promoter region bound by the trimeric RNA-dependent RNA polymerase complex formed by the viral proteins polymerase basic protein 1 (PB1), polymerase basic protein 2 (PB2), and polymerase acidic protein (PA) [1]. The rest of the vRNA is encapsidated by several copies of the nucleoprotein (NP) to form viral ribonucleoproteins (vRNPs) that constitute independent units for viral transcription and replication [2]. In the late steps of viral replication, newly synthesized vRNPs are exported from the nucleus and transported towards the apical plasma membrane where they are incorporated into budding viral particles. Current knowledge about nuclear export and cytoplasmic transport of the vRNPs to reach budding sites is reviewed in [3].

In order to be replication-competent and fully infectious, IAV particles must incorporate at least one copy of each of the eight gene segments. Electron microscopy (EM) of IAVs repeatedly revealed that vRNPs were arranged in a “7+1” manner in budding virions, with a central vRNP surrounded by seven others [4,5]. The genome of IAVs was found to be haploid with equimolar levels of each segment inside viral particles [6,7]. It is now well established that genome packaging (i.e., the incorporation of a set of eight distinct vRNPs into budding viral particles), follows a specific rather than a random pathway, guided by segment-specific *cis*-acting packaging signals on each vRNA [8,9]. The existence of these packaging signals was initially inferred from the occurrence of defective interfering RNAs (DI RNAs) derived from IAV gene segments (for reviews see [8,9]). Such RNAs carry large deletions of the central ORFs but systematically preserve the NCRs and the adjacent 3′ and 5′ ends of the coding region, are efficiently replicated, and compete with their parental vRNAs for packaging into the progeny viruses. More recently, the development of reverse genetics (RG)—i.e., the generation of recombinant influenza viruses from cloned cDNAs (for a detailed review, see [10])—has been key to the understanding of various aspects of the biology of IAVs, including packaging of the viral genome. Expression of the eight viral or viral-like RNAs was achieved from a set of plasmids containing RNA polymerase I (PolI) promoter sequences and either a PolI terminator or a ribozyme sequence that generate the correct 5′ and 3′ ends, respectively. These plasmids were transfected into human embryonic kidney cells expressing the simian virus 40 (SV40) large T antigen (HEK293T) along with expression plasmids under the control of the RNA polymerase II (PolII) promoter for, at minima, the synthesis of the viral PB1, PB2, PA and NP proteins, which results in the reconstitution of vRNPs and allows the initiation of a viral replication cycle [11,12]. Alternatively, a set of eight bidirectional pPolI-pPolII plasmids has been used [13].

In this article, we review the diverse experimental approaches available to map the *cis*-acting packaging signals on individual vRNAs and to understand how they function to promote the assembly and packaging of a set of eight distinct vRNAs into viral particles.

## 2. Mapping of *cis*-Acting Packaging Signals on Individual vRNAs

**Figure 2:**
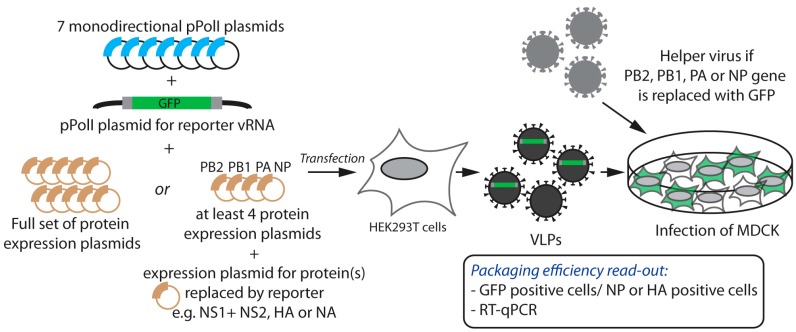
Experimental strategy to study packaging of reporter vRNAs into virus-like particles (VLPs). Human embryonic kidney cells expressing the simian virus 40 (SV40) large T antigen (HEK293T) were co-transfected with the indicated pPolI and expression plasmids, including a pPolI plasmid for expression of the GFP-reporter vRNA (see Figure 1). The grey boxes surrounding the GFP correspond to the portions of coding regions under investigation by deletional analysis. Supernatants containing the VLPs were used to infect, for instance, Madin-Darby canine kidney (MDCK) cells. The reporter gene may or may not be incorporated into VLPs, together with the seven other segments. When the reporter replaced one of the four proteins involved in formation of the vRNPs, helper viruses of a different genetic background (grey) were usually delivered in *trans* to provide those functional proteins in the infected cells. The incorporation efficiency of the vRNA of interest was calculated as the ratio of the number of GFP-positive cells (i.e., the VLPs containing the vRNA of interest) to the total number of nucleoprotein (NP)- or hemagglutinin (HA)-positive cells (i.e., the total number of infectious VLPs) while quantitative reverse transcription polymerase chain reaction (RT-qPCR) allowed quantification of all vRNA segments. PB1: polymerase basic protein 1; PB2: polymerase basic protein 2; PA: polymerase acidic protein; NA: neuraminidase; NS1: non-structural protein 1; NS2: non-structural protein 2.

**Figure 3:**
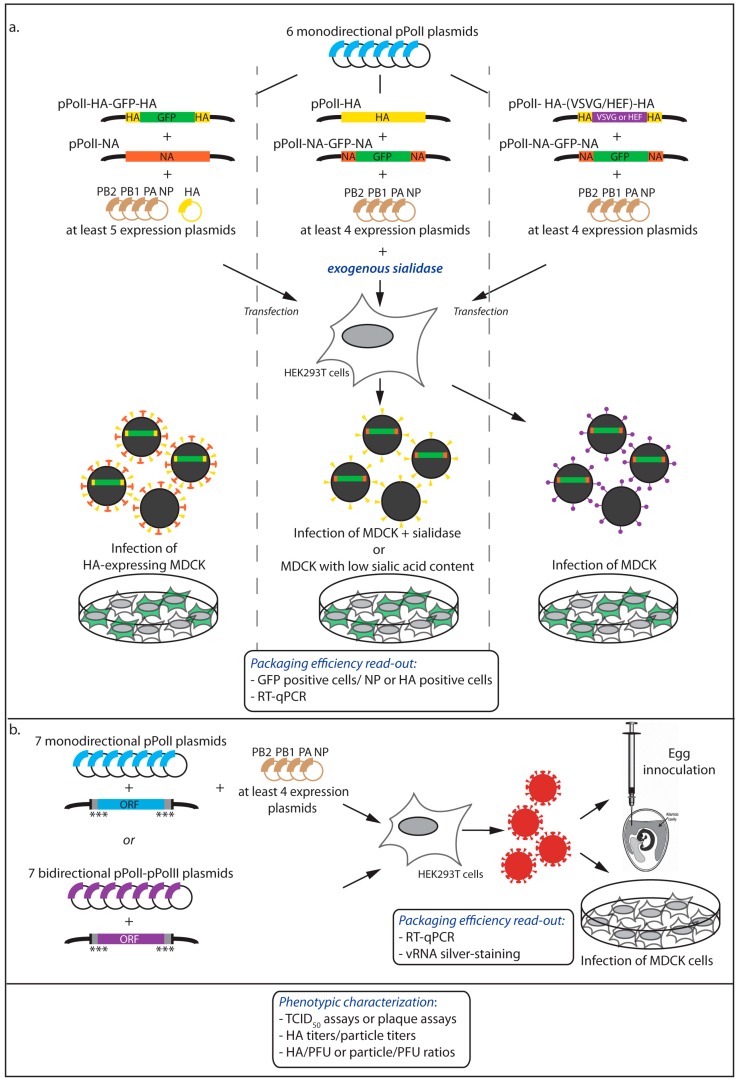
Experimental strategies to study packaging of engineered vRNAs into replication-competent viruses. (**a**) Incorporation of reporter vRNAs into replication-competent viruses. HEK293T cells were co-transfected with the indicated pPolI and expression plasmids, including a plasmid carrying a GFP gene flanked by the HA (left panel) or NA (middle panel) packaging sequences. In the absence of functional HA, HA-GFP-HA viruses can be rescued upon expression of HA in transfected cells and grown efficiently in MDCK cells that stably express HA (left panel). NA-GFP-NA viruses were produced and grown efficiently in cells supplemented with exogenous sialidase or with low sialic acid content (middle panel). Viruses possessing the vesicular stomatitis virus G (VSVG) protein or the hemagglutinin/esterase/fusion (HEF) protein of an influenza C virus as the surface protein instead of HA and NA were produced by co-transfection of HEK293T cells with six monodirectional pPolI plasmids together with the pPolI-HA-(VSVG/HEF)-HA and pPolI-NA-GFP-NA plasmids (right panel). In all cases, viral particles may contain or not the reporter gene, together with the seven other segments. The incorporation efficiency of the vRNA of interest was calculated as described in the legend of Figure 2; (**b**) Incorporation of vRNAs carrying point mutations into replication-competent viruses. HEK293T cells were co-transfected with the indicated pPolI and expression plasmids, or bidirectional pPolI-pPolII plasmids, including a pPolI or pPolI-pPolII plasmid for expression of the vRNA of interest carrying mutations in the NCRs or synonymous mutations (indicated by asterisks) in the termini of the coding sequence. Supernatants were used to infect MDCK cells and/or to inoculate embryonated chicken eggs, the latter being usually performed with the egg-adapted influenza A/Puerto Rico/8/34 (PR8) virus. The vRNA content of viral particles was quantified by RT-qPCR or by densitometry on silver-stained denaturing polyacrylamide gels (for egg-grown viruses). Phenotypic assays of rescued viruses were used as an indicator of the disruptive effect of deletions (**a**) or mutations (**b**) within vRNAs on viral growth. TCID_50_: median tissue culture infective dose; PFU: plaque-forming units.

Packaging signals were initially coarsely mapped by deletional analysis, and then further defined by directed mutagenesis. Upon introduction of deletions or mutations (Figure 1b,c) into a reverse genetics pPolI-driven plasmid, the efficiency with which engineered vRNA molecules were incorporated either into virus-like particles (VLPs) (Figure 2) or into replication-competent infectious viruses (Figure 3), was measured. Since RNA molecules adopt functionally important secondary and tertiary structures, rationalizing mutagenesis in order to maintain structural features is problematic. Short local secondary structure elements have been predicted for IAV vRNAs [14,15] and only recently, the secondary structure of a non-structural (NS) gene segment vRNA was inferred from the combination of experimental data and computer folding [16]. However, naked (protein-free) RNA was used while it is likely that the secondary structure of IAV vRNAs is heavily influenced by the encapsidation with NP and the binding of the polymerase [17]. An additional difficulty lies in the fact that packaging signals extend to the coding sequences and mutagenesis therefore requires the introduction of synonymous mutations in the ORFs. Interestingly, sequences important for packaging were generally found to be at least partly conserved, leading to a new approach for rationalized mutagenesis based on the search for regions containing clusters of codons with a lower frequency of synonymous mutations than expected from amino acid conservation or codon bias [18,19].

### 2.1. Incorporation of Reporter vRNAs into VLPs

Partial replacement of the vRNA sequences by a reporter gene, typically GFP (Figure 1b), allows the detection of the reporter vRNA upon incorporation into a VLP and subsequent delivery into a host cell (Figure 2). Kawaoka’s group pioneered this single-cycle infectious virus approach with reporter HA (HA-GFP-HA) [20] or NS (NS-GFP-NS) vRNAs [21] where the GFP coding sequence was flanked by the NCRs and portions of various length of the coding region derived from both termini (Figure 1b). HEK293T cells were co-transfected with a pPolI plasmid that drove the expression of the reporter vRNA, together with the seven other pPolI plasmids and at least the four expression plasmids for the PB1, PB2, PA, and NP proteins plus the one for the protein(s) replaced by the reporter gene. The supernatants containing the VLPs were used to infect, for instance, MDCK cells, and the incorporation efficiency of the vRNA of interest was calculated as the ratio of the number of VLPs containing the vRNA of interest (i.e., the number of GFP-positive cells) to the total number of infectious VLPs, commonly assessed by the number of NP- or HA-positive cells detected upon immunostaining and cell count analysis (Figure 2).

To estimate the incorporation efficiency of vRNAs encoding one of the three polymerase subunits or the nucleoprotein, the system has to be adapted. Indeed, each of these proteins is needed for the expression of the reporter gene upon infection with the VLPs, and therefore it has to be provided in *trans*. One option has been to co-infect MDCK cells with the VLPs and a helper virus [22,23]. Then, to accurately determine the total number of infectious VLPs, it was necessary to use specific HA and/or NP antibodies that do not recognize the proteins derived from the helper virus (Figure 2). A different approach was used by Liang et al., who co-transfected HEK293T cells with the pPolI reporter plasmid together with eight bidirectional pPolI-pPolII plasmids, hence producing VLPs as well as wild-type helper virus in the supernatant [24]. However, under this particular setting, analysis of the results is complicated by the fact that the reporter vRNA competes with the wild-type vRNAs for incorporation into virions.

### 2.2. Incorporation of Engineered vRNAs into Replication-Competent Viruses

In the context of replication-competent viruses, deletional analysis with a reporter gene is possible only with genes that can be compensated for, typically HA and NA (Figure 3a). For example, recombinant viruses carrying a GFP gene flanked by the HA packaging sequences have been rescued upon expression of HA in transfected cells and grown efficiently in MDCK cells that stably express the HA glycoprotein during multiple passages [25] (Figure 3a, left panel). Likewise, a segment containing a GFP gene flanked by the NA packaging sequences has been incorporated and stably maintained during viral replication in cells supplemented with exogenous *Vibrio cholerae* sialidase [26] or in cells with poor content in sialic acids [27] (Figure 3a, middle panel).

It is also feasible to generate viruses possessing a surface glycoprotein that substitutes for both HA and NA functions. This was achieved using the vesicular stomatitis virus G (VSVG) protein by Kawaoka and co-workers [20] and using the hemagglutinin/esterase/fusion (HEF) protein of an influenza C virus by Palese and co-workers [28]. The VSVG or HEF gene were introduced instead of the HA coding sequence and flanked by the HA packaging sequences. Such chimeric viruses can therefore express GFP introduced as an eighth segment, to map the NA packaging sequences (Figure 3a, right panel).

When the packaging efficiency is assessed in an infectious context, the choice of the reporter gene is not neutral. Differences were observed between the HA-VSVG-HA and the HA-GFP-HA viruses in terms of minimal sequences required for efficient incorporation into viral particles [25]. This is likely due to the fact that the VSVG protein was required for attachment and infection and therefore the packaging of the corresponding segment was, unlike HA-GFP-HA, under positive selection. To determine whether the length of the vRNA or the sequence of the reporter had any effect on the packaging, constructs with a single GFP or red fluorescent protein (RFP), or with a tandem of GFP genes were compared [25]. Although the packaging of all constructs was efficient, the HA-GFP-GFP-HA segment, whose length is very close to the HA vRNA length, showed a higher level of incorporation compared to HA-GFP-HA. It is noteworthy that the virus with the HA-RFP-HA segment could replicate efficiently only when expression of RFP was lost due to non-sense mutations.

Mutational analysis was used to map more precisely the nucleotides that form the packaging signals [19,21,25,29,30,31] (Figure 1c and Figure 3b). Using reverse genetics, the effect of mutations has been looked at broadly by phenotypic characterization of the viruses, or more specifically by quantitative measurement of the viral RNA content. Analyses of this kind also apply to recombinant viruses carrying a reporter vRNA as described above.

#### 2.2.1. Phenotypic Characterization of the Viruses

The properties of the wild-type and mutant viruses have also been compared by assessing the production of infectious virus particles by median tissue culture infective dose (TCID_50_) assays or plaque assays. The plaque phenotype is also a marker of the replicative properties of a given virus and has been used as an indicator of the disruptive effect of mutations on viral growth [29,30]. Hemagglutination titers or particle titers obtained by electron microscopy counting of virions were used to establish HA/plaque forming units (PFU) or particle/PFU ratios, which helps evaluating whether the mutations are detrimental or not. Mutants that have a disruptive effect on packaging usually display an increased particle/PFU ratio as compared to the wild-type [29]. It is noteworthy that typically observed reductions in viral loads ranged from 1 to 3-logs when grouped point mutations were tested but never completely abolished the rescue of infectious viruses. This could be due to the discontinuous nature of the packaging signals along a particular gene segment [18,19,25,29,30,31], allowing the loss of certain packaging sequences to be compensated for by others. In addition, the proposed plasticity in the positioning of the eight vRNPs within virions [32,33] most likely also increases the chances of correct genome packaging.

#### 2.2.2. Quantitative Measurement of the Viral RNA Content

The most frequently used method to directly analyze packaging efficiency has been quantification by quantitative reverse transcription polymerase chain reaction (RT-qPCR) of each of the genomic segments extracted from purified viral particles [19,29,30], which offers the possibility to analyze not only the packaging efficiency of the mutated vRNA gene but also that of the other vRNAs (see below). However, in the absence of standard curves obtained from synthetic vRNAs, only relative concentrations of RNA can be determined. RT-qPCR data need to be (i) normalized to the total vRNA amount by equalizing the level of one reference segment; and (ii) compared to the wild-type virus [19,25]. The latter point makes the assumption that in the wild-type virus, each vRNA is detected in equimolar ratio. This may not be the case if, for instance, defective vRNAs are not detected by the RT-qPCR and vary in proportion from segment to segment. The NA segment has commonly been used as a reference segment, because its packaging appears relatively less constrained compared to other segments [19,22,34]. When RT-qPCR data are normalized by comparison with standards, segment/particle ratios can be calculated, using matching particle titers, and compared to the ratios obtained for the wild-type virus; plaque titer values can also be used to calculate segment/PFU ratios [29,30]. These are useful parameters, as mutations in a given vRNA can decrease the overall packaging of all vRNA segments while maintaining equimolar ratios, as shown by Hutchinson and co-workers for the matrix (M) gene segment vRNA [29].

As an alternative to RT-qPCR analysis, the vRNA content of viral particles has been quantified on silver-stained denaturing polyacrylamide gels followed by densitometry. This method usually requires egg-grown viral stocks, in order to get sufficient amounts of vRNAs that should be extracted from equal numbers of viral particles (preferably physical particles) [29,30,34]. In addition, the accuracy of densitometry data is subjected to the existence of a linear relationship between the staining intensity and the amount of nucleic acid that needs to be verified for the concentration range used in the experiment [29].

### 2.3. Relevant Experimental Controls

Whether VLPs or replication-competent viruses are used to investigate vRNA incorporation efficiencies, various assays need to be performed to rule out possible mutation-induced pleiotropic effects that could provoke defects at earlier stages of the virus life cycle. Typically, the levels of reporter or mutant vRNAs in plasmid-transfected cells have to be quantified, usually by RT-qPCR (alternatively by primer extension or Northern blotting), to ensure that equivalent amounts of wild-type and engineered RNAs are being transcribed and are available in infected cells for packaging into virions [19,22,30,31]. Likewise, the amounts of reporter protein produced in transfected cells have to be monitored, to control for potential bias at the post-transcriptional level [22,31]. Wild-type and mutant viruses can also be investigated for potential defects in trafficking by fluorescence in situ hybridization (FISH), possibly combined with simultaneous labeling of the nuclear envelope, the plasma membrane, or other sub-cellular compartments by indirect immunofluorescence.

## 3. Unraveling the Mechanism for Co-Packaging of Eight Distinct vRNAs

Interestingly, the mutational approach described above has revealed that reduction in incorporation of a particular mutated vRNA was also accompanied, in some cases, by a decreased incorporation efficiency of other vRNAs [19,22,25,29,30], strongly suggesting the existence of intersegment interactions that may drive the assembly process. In the particular case of NP vRNA, it was shown that the NCRs guarantee incorporation of the vRNA into virus particles while the previously defined packaging signals (including the NCRs and the termini of the coding sequence) ensure incorporation of a correct set of vRNPs [35]. Interestingly, the HA NCRs, and more specifically the 3′ NCRs, found to be subtype specific and of varying length and sequence, strongly modulated virus replication by impacting the level of HA vRNA incorporation, without significantly reducing incorporation of the other vRNAs [36]. Altogether, these data suggest that the NCRs are critical for virion selective incorporation of vRNAs, whilst nucleotides in the coding regions could be involved in intersegment interactions. In addition, packaging also appears to be a hierarchical process as not all vRNAs are equally important for efficient genome incorporation: PB2 vRNA is more important than PB1 and PA vRNAs in the influenza A/WSN/33 strain [22] while PB2, M, PA, and NP vRNAs play a more important role than the remaining four vRNAs in the packaging process for the influenza A/Puerto Rico/8/34 (PR8) strain [19,30,34]. A recent study suggests that sequential formation of vRNP sub-bundles (an assembly of less than eight vRNPs) within the cytoplasm might be required for efficient packaging of a full set of eight distinct vRNPs [37]. However, it is still unclear whether the “genome bundling” process is driven by the genome packaging sequences (for details, see [3]).

### 3.1. Co-Packaging Assays

#### 3.1.1. The Rewiring Approach

To study co-packaging, Palese and co-workers exploited a seven-segmented chimeric PR8, with no NA segment, but with a segment encoding the HEF protein of an influenza C virus, flanked by HA packaging sequences [28]. This virus was used to study the role of the PB1, PB2, PA, NP, M, or NS segment in genome packaging, by generating (i) a set of seven-segmented viruses in which one of the gene-specific packaging sequences was mutated and replaced functionally with the NA packaging sequences, leading to a “rewired segment”; and (ii) a matched set of eight-segmented viruses with an additional gene segment carrying the GFP ORF surrounded by the wild-type packaging sequences of the PB1, PB2, PA, NP, M, or NS segment [34]. Comparison of the viral growth between seven-(GFP minus) and eight-(GFP plus) segmented viruses was one of the readouts performed to assess the role of the packaging sequences of a particular vRNA segment: impairment of viral growth for the seven-segmented virus but rescued for the eight-segmented virus is an indicator of the importance, for global packaging, of the gene-specific incorporation sequence that has been targeted.

#### 3.1.2. Competitive Reverse Genetics

**Figure 4:**
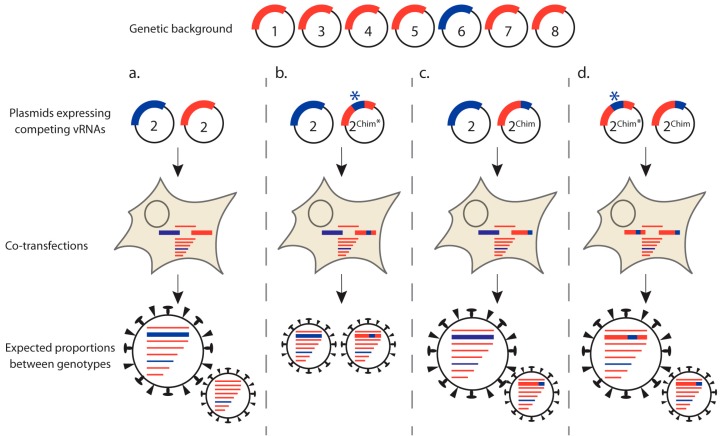
Nine-plasmid-based competitive reverse genetics (RG) as a tool to analyze vRNA-vRNA co-packaging. In nine-plasmid competitive RG experiments, HEK293T cells were co-transfected with seven bidirectional pPolI-pPolII RG plasmids defining the genetic background and two plasmids that express competing vRNAs. (**a**) If two vRNAs (for instance vRNAs 2 and 6, in blue) were found to co-segregate in an otherwise different genetic background (red) during a genetic reassortment experiment, the “2-blue:6-blue:other-red” genotype is expected to emerge predominantly during a nine-plasmid competitive RG experiment; (**b**) Chimeric constructs (of vRNA 2 for instance) can be used to delineate the region of the vRNA involved in guiding co-packaging of the two vRNAs. If the region marked by an asterisk (2^Chim*^) is the important one for the co-packaging interaction with the vRNA partner (6, in blue), the wild-type and chimeric genotypes are expected to emerge with approximately the same frequency; (**c**) A chimera carrying a region of vRNA 2 that is not important for co-packaging of the two partner vRNAs (2^Chim^) is expected to emerge at much lower frequency than the wild-type; (**d**) Finally, competition between the two chimeras is expected to be in favor of the one bearing the region involved in co-packaging of the two vRNA partners.

The segmented nature of the IAV genome drives the need for a sophisticated and specific packaging process but also provides the virus with a mechanism to facilitate the exchange of intact gene segments, in a process named “genetic reassortment”, when two distinct influenza viruses co-infect the same cell [38]. Alternatively, genetic reassortment can occur after plasmid transfection using two full sets of RG plasmids for each virus. The genetic reassortment process is clearly biased as all possible gene combinations (256 when two different viruses co-infect the same cell) are not observed under natural or experimental conditions and certain gene segments tend to co-segregate [39,40,41,42,43]. Yet, some rare reassortant genotypes can efficiently be rescued by RG with a defined set of eight plasmids to transfect. Hence, in an intermediate approach, competitive plasmid transfection with more than eight (but less than 16) RG plasmids is a tool that has been exploited to investigate co-segregation and co-packaging of pairs of vRNAs (Figure 4) [43].

The combined use of nine-plasmid reverse genetics and site-directed mutagenesis to construct chimeric functional gene segments has revealed molecular determinants that drive co-segregation of pairs of vRNAs ([43] and Gilbertson et al., this issue) (Figure 4). For instance, if precise vRNA/vRNA interacting sequences have been delineated, *trans*-compensatory mutants, that need to be silent if located in the coding sequence(s), can be designed on both RNA partners and tested in four competitive RG assays, where the wild-type and mutant counterpart for one vRNA compete for incorporation with either their wild-type or mutant vRNA partner, and vice versa. If the genotype of resulting viral particles is strongly biased towards either a wild-type or a double compensatory mutant, this is a strong indication that vRNAs that interact together are preferentially co-packaged [9,44]. Generally, competitive RG can become a tool to identify associations between vRNAs that are important for co-packaging during viral budding and/or co-segregation during genetic reassortment.

A limitation of genetic reassortment as a tool to analyze packaging lies in the fact that protein compatibility can constrain the reassortment process at stages of the viral life cycle other than packaging. Therefore, the predominance of a given gene combination can occur not because of differences in co-packaging efficiency but because of differences in fitness of the corresponding reassortant viruses. Another limitation lies in the relatively small number of viral clones that can be analyzed to date in order to generate statistically significant results. Indeed, the identification of reassortant viruses arising from RG experiments requires genotyping of clonal isolates. Classically, vRNAs are extracted from plaque-purified viruses and reverse transcription-PCR (RT-PCR) products are sequenced [45]. Standard or quantitative PCR can also be performed taking advantage of sequence differences between the two parental strains if they are heterologous enough. A new method for genotyping reassortant viruses was recently described, based on the differential melting properties of double-stranded DNA (dsDNA) PCR amplicons with differences in sequence as little as 1 nt. This high-resolution melting (HRM) analysis was based on the design of primers that bind to conserved regions in both strains of interest and surround 50 to 150 nt-long regions that contain one or more sequence differences between both strains [46]. Compared to the previous approach, HRM may allow discrimination between segments that are very similar. On the other hand, highly divergent segments such as HA and NA may not contain enough conservation to allow annealing of the primers to identical regions in both strains. In the particular case of a mixed population of viruses arising from a defined nine-plasmid reassortment experiment as shown in Figure 4, deep sequencing targeting the competing segments of interest has also been used to give the relative abundance of the two competing genotypes [47]. Pyrosequencing, which can provide quantitative data, is another alternative [48].

### 3.2. Visualization of vRNP Transport and Bundling

#### 3.2.1. Single-Molecule Fluorescence In Situ Hybridization (smFISH)

Like all hybridization-based techniques, FISH detects RNA molecules using fluorescently labeled probes that are complementary to the sequence of interest. Single-molecule sensitivity can be achieved by using several probes targeting different regions of the same RNA. Between 15 and 48 oligonucleotide probes labeled with a single fluorophore are usually used to probe one RNA species.

The smFISH technology has been applied to surface-immobilized viral particles to estimate the co-packaging efficiency of two different vRNA segments into viruses, using for instance a mixture of Cy3- and Cy5-labeled probes against two different RNA segments [49]. The copy number of each vRNA segment being packaged was also evaluated upon photobleaching analysis [49].

The smFISH technique was also applied to the detection of vRNAs within infected cells, using for instance two-color [50] or four-color smFISH [37] for the visualization of two or four distinct vRNA segments, respectively. These approaches gave insights into the trafficking of pre-formed vRNA complexes from the nucleus to the plasma membrane and are discussed in detail in [3]. Four-color smFISH probes have been used to test all pair-wise combinations of vRNA segments [37]. Combining smFISH with immunostaining (or expression of a protein fused to a fluorescent protein) allowed quantification of the proportion of co-localized vRNAs that are associated with a cellular or a viral protein [37,50].

Importantly, specificity of smFISH probes needs to be verified on cells expressing a single vRNA transcript from a pPolI expression plasmid. The distance threshold that defines co-localization needs to be defined based on the distances between fluorescent spots measured with a positive control (two labeled probes targeting two regions of the same vRNA molecule) and a negative control (two labeled probes targeting non-interacting RNA molecules). On a standard microscope this threshold is usually set around 250 nm, which does not allow to unambiguously conclude that two vRNAs are physically interacting with each other. Given the high density of vRNAs in infected cells, it may be difficult to distinguish actual physical interactions from crowding-induced random proximity. Combining smFISH analysis with fluorescence resonance energy transfer (FRET) or with super-resolution microscopy [51] could help to overcome this limitation.

The sensitivity and quantitative accuracy of smFISH are also limited. The fact that 100% of co-localization for probes targeting the same vRNA cannot be achieved [49,50] may be explained by intrinsic defects of some vRNAs or by the limit of detection of co-localized spots. The quantification of the copy number of vRNA packaged within influenza virions may be overestimated by self-aggregation of virus particles (10% of the spots exhibit high number of photobleaching steps) [49]. Finally, visualization of vRNA segments has been so far restrained to two- or four-color smFISH due to the limited availability of fluorophores with non-overlapping spectra, thus hindering assessment of the localization and interactions between more than four vRNA segments within a single cell. Multiplex smFISH has been achieved by sequential barcoding, where each segment is detected multiple times through hybridization, imaging, and probe removal cycles, and appears in a different color during each cycle [52,53].

#### 3.2.2. Electron Microscopy and Tomography

Visualization of budding virions in traverse thin-section by EM has repeatedly revealed that the eight vRNPs are arranged in a “7+1” manner [5,29,32,33,44]. Cross-sections of budding viral particles were analyzed by EM to investigate their vRNP content and the ratio of full, partially full or empty viral particles was determined. Such visualization provided a global qualitative analysis of the vRNP content of influenza virions and has been used to assess global packaging defects of mutant viruses compared to their wild-type counterpart [54]. The shortcomings of this method are the likelihood that viral particles are sectioned below the longest vRNPs and will thus appear empty, the fact that the different segments cannot be distinguished from each other, and that, for yet unknown reasons, the “7+1” architecture is rapidly lost after budding. The analysis of a large number of different cross-sections is one way to partly correct for these biases.

The arrangement of vRNPs inside influenza A viral particles has also been analyzed by cryo-electron tomography, which allows to reconstruct the 3D structure of individual ice-embedded viral particles. Cryo-electron tomography confirmed that influenza virions most commonly contain eight vRNPs that form a near-parallel bundle where the “7+1” configuration can be observed, with the length of vRNPs varying from 24 to 110 nm [4]. Based on the fact that differences in length allow to distinguish most of the vRNPs inside the viral particle, the relative arrangements of vRNPs with respect to each other was studied [32,33]. Identification of packaged vRNAs could be envisaged with specifically engineered, easily distinguishable, vRNA segments. The combination of scanning transmission electron microscopy (STEM) tomography and immuno-EM was also used to assess the orientation of vRNPs within virions [55].

### 3.3. In Vitro vRNA-vRNA Interactions Assays

Since the first demonstration of the existence of segment-specific packaging signals by RG, direct interactions between vRNAs appeared as an attractive hypothesis to explain the selective packaging mechanism. This hypothesis is reinforced by the fact that no viral or cellular protein specifically recognizing an IAV segment-specific packaging signal has been identified so far. Furthermore, inside vRNPs, vRNAs would be largely exposed on the external surface of NP oligomers [17,56,57], allowing such RNA/RNA interactions to occur. However, demonstrating the existence of specific vRNA-vRNA interactions in viral particles without prior knowledge of the sequences involved is challenging.

To address this question, in vitro assays have been used as a tool to identify interacting RNA partners. The vRNAs were synthesized by in vitro transcription, and the interactions between all possible pairs of vRNAs analyzed using an electrophoretic mobility shift assay. Using such assays, single and strain-specific interaction networks maintained by sequence-specific RNA-RNA interactions were identified for two viral strains [32,44]. The interacting sequences between two particular vRNAs were precisely delineated, in vitro, by a combination of deletion analysis, antisense oligonucleotide mapping and bioinformatics prediction of interacting sequences [44,54]. To confirm the existence of the interaction in vitro, *trans*-complementary mutants on both vRNA were designed and tested. This strategy, however, has its limitations as it is unknown whether all in vitro interactions take place within the viral particles and conversely, whether all interactions that occur in virions can be identified in vitro. Therefore, the relevance of in vitro identified vRNA-vRNA interactions and their importance for selective co-packaging needs to be validated in an infectious context. To do so, the phenotypic features (see Section 2.2) of the wild-type virus must be compared with those of each single mutated virus (i.e., viruses bearing disrupting mutations in only one of the partner vRNAs), and with those of a double-mutant virus bearing the *trans*-complementary mutations that restore the interaction [54]. Packaging of vRNA can also be quantified directly by RT-qPCR (see Section 2.2) while the importance of intersegment interaction to guide vRNA co-packaging can be tested by competitive RG (see Section 3.1).

## 4. Conclusions

Reverse genetics has been extensively used to engineer IAVs in order to investigate the mechanisms underlying packaging of their genome. Over the past ten years, remarkable progress was made in understanding this process, based on a combination of genetic, imaging, and biochemical complementary approaches. Recent data suggest that vRNPs could form sub-bundles in the cytoplasm of infected cells whilst transported towards the plasma membrane in association with Rab-11-positive recycling endosomes (reviewed in [3]). In addition, the large set of data available strongly suggests that subsequent packaging at the viral budding site is driven by the formation of strain-specific supra-molecular complexes of eight distinct vRNPs held together by strain-specific vRNA-vRNA interactions.

However, to confirm this model and further elucidate the molecular mechanisms involved, the sensitivity, resolution, quantitative accuracy and/or statistical power of the methods being applied need to be improved. Expanding the observations to various IAV strains including the novel bat IAVs [58] and to influenza B [59] and C viruses will also help to get a comprehensive view of this complex and versatile process. Work on other viruses with a segmented genome could also provide valuable information. Interestingly, packaging of the reoviruses’ 11-segmented double-stranded RNA genome is also believed to be sequential and mediated by RNA-RNA interactions [60]. An identical workflow as the one described above for IAV, starting with in vitro identification of RNA-RNA interactions up to the validation of the role of the interaction for efficient viral replication, was recently used to decipher the packaging mechanism of Bluetongue virus [61].

Understanding the fundamental molecular mechanisms that underlie packaging of the IAV genome is of the utmost importance since it should lead, in the long term, to a better understanding of the mechanisms sustaining genetic reassortment of IAVs [9,38] and help to assess the likelihood of the appearance of reassortant viruses, including those with pandemic potential. It might also help to manipulate the genetic reassortment process in order to improve the generation, selection, and production of high yielding vaccinal seeds [62].
